# When Ethical Leadership Is Not Enough: A Person-Centred Analysis of Employee Strain and Turnover Intentions

**DOI:** 10.3390/bs16091530

**Published:** 2026-08-31

**Authors:** Cemal Zehir, Umut Elbir, Melike Artar Bıyıklar, Yavuz Selim Balcıoğlu

**Affiliations:** 1 Department of Business Administration, Faculty of Economics and Administrative Sciences, Yıldız Technical University, Istanbul 34220, Turkey; czehir@yildiz.edu.tr; 2 Eurasian Economic International Scientific Research Center, Azerbaijan State University of Economics, Baku 1001, Azerbaijan; 3 Department of Property Protection and Security, Vocational School of Health Services, Istanbul Bilgi University, Istanbul 34387, Turkey; umut.elbir@bilgi.edu.tr; 4 Department of Business Administration, Faculty of Business Administration, Gebze Technical University, Gebze 41400, Turkey; 5 Department of Management Information Systems, Faculty of Economics and Administrative Sciences, Dogus University, Istanbul 34775, Turkey; ysbalcioglu@dogus.edu.tr

**Keywords:** ethical leadership, job stress, turnover intention, perceived organisational support, latent profile analysis, person-centred research

## Abstract

Ethical leadership is usually examined through its consequences for employees, and it is often assumed that employees who rate their supervisor as ethical will also report lower strain and weaker intentions to leave. This study tests that assumption using a person-centred design. Survey data were collected from 247 shift-working employees of a single Turkish manufacturing organisation. Participants rated the ethical leadership of their immediate supervisor and reported their own job stress, perceived organisational support, turnover intention and career planning. Latent profile analysis identified four subgroups. Two of these are of particular interest: both report high ethical leadership and organisational support yet differ sharply in job stress (4.37 vs. 2.48) and turnover intention (4.10 vs. 2.18). This divergence helps explain why job stress is essentially unrelated to ethical leadership (r = 0.02, *p* = 0.820) and to perceived organisational support (r = 0.04, *p* = 0.492) at the bivariate level, while remaining strongly associated with turnover intention (r = 0.43, *p* < 0.001). Favourable evaluations of a supervisor therefore appear compatible with very different experiences of strain. Because the design is cross-sectional and all measures are self-reported by the same source, the findings describe patterns of association rather than causal processes. The results suggest that leadership-focused interventions alone may not address all sources of strain-related turnover intention and that job demands require separate assessment.

## 1. Introduction

Ethical leadership is generally associated with favourable employee outcomes, including greater trust, engagement, and commitment and lower withdrawal intentions (Brown & Treviño, 2006; Treviño et al., 2000). This largely beneficial account, however, leaves less room for the possibility that employees who evaluate their supervisors positively may nevertheless experience substantial strain or consider leaving the organisation. A favourable leadership environment and a favourable employee experience are not necessarily equivalent. Employees may evaluate their supervisors favourably while simultaneously experiencing substantial job stress or considering leaving the organisation (Palanski & Yammarino, 2009). This possibility raises a question that has received less attention in ethical leadership research: whether positive evaluations of ethical leadership are accompanied by similarly positive employee experiences across the workforce, or whether distinct configurations of leadership perceptions, organisational support, strain, and withdrawal coexist within the same organisational setting.

Emerging evidence complicates the assumption that favourable perceptions of ethical leadership are uniformly accompanied by positive employee experiences. Ethical leaders may promote fairness, integrity, and accountability, yet the standards and expectations associated with such leadership do not necessarily eliminate the demands employees encounter in their work (De Hoogh & Den Hartog, 2008). Studies in healthcare settings, for example, suggest that favourable leadership evaluations may coexist with turnover intention while burnout remains independently associated with employees’ intentions to leave even when leadership is evaluated positively (Lambert et al., 2024). These findings point to an important distinction: employees’ evaluations of their supervisors and their own experiences of strain represent related but distinct aspects of organisational life (Yue et al., 2022). Positive perceptions of ethical leadership may therefore coexist with substantial job stress or withdrawal intentions, raising the possibility that the benefits associated with ethical leadership are not experienced uniformly across employees.

Perceived organisational support adds a distinct organisational-level evaluation to this picture (Eisenberger et al., 1986). Whereas ethical leadership captures employees’ evaluations of their immediate supervisor, POS reflects the extent to which employees believe that the organisation values their contributions and cares about their well-being (Kaluza et al., 2022). Examining these perceptions jointly therefore allows us to ask whether favourable supervisory and organisational evaluations form uniformly beneficial employee experiences or occur within more heterogeneous configurations of strain and withdrawal (Spence, 1978).

This is a question about the distribution of experiences within a workforce rather than about the average level of any single variable, and it therefore calls for a person-centred method (Rodgers et al., 2024). Variable-centred approaches estimate average associations across a sample and may therefore obscure qualitatively different configurations of employee experiences. A person-centred approach such as latent profile analysis (LPA) instead allows subgroups of employees characterised by distinct combinations of leadership perceptions, organisational support, strain, and withdrawal to emerge from the data (Bauer, 2022). This distinction is particularly relevant when favourable leadership evaluations may coexist with different levels of stress or turnover intention across employees (Gillet et al., 2019). Where two subgroups share a high level on one variable but diverge on another, LPA will separate them while regression will not. This capacity to detect configurations that cancel out in aggregate is the specific reason for adopting the method here, rather than a general preference for person-centred analysis.

The present study applies LPA to survey data from 247 shift-working employees of a single manufacturing organisation. Employees rated the ethical leadership of their immediate supervisor and reported their own job stress, perceived organisational support, turnover intention and career planning. Career planning was included as an exploratory indicator capturing the extent to which employees perceive a developmental future for themselves within the organisation. The study addresses three questions:

(1) How many distinct profiles of employee-rated ethical leadership, job stress, perceived organisational support, turnover intention and career planning can be identified in this workforce?
(2) Do any of these profiles combine favourable evaluations of supervisory ethical leadership with high levels of job stress or turnover intention?
(3) How do the profiles relate to demographic and occupational characteristics?

These are stated as research questions rather than hypotheses. Directional hypotheses require an a priori specification of the number of profiles and their content, and no prior study has estimated a profile solution on this combination of variables in a manufacturing workforce. Enumerating profiles is an inductive task in which the number of subgroups is itself an empirical result rather than a parameter to be tested (Bauer, 2022). Formulating hypotheses about profiles that have not yet been shown to exist would give the analysis a confirmatory appearance it does not have. The associations examined in the third question are correspondingly exploratory.

## 2. Literature Review

### 2.1. Ethical Leadership and Employee Perceptions

Ethical leadership refers to leaders’ demonstration of normatively appropriate conduct through their personal actions and interpersonal relationships, as well as their promotion of such conduct among employees through communication, decision-making, and reinforcement processes (Brown & Treviño, 2006). Because ethical leadership is manifested through observable behaviours such as fairness, integrity, accountability, and concern for others, it is conceptualised not merely as an individual characteristic of the leader but as a relational form of leadership that employees evaluate through their everyday work experiences (Lambert et al., 2024). Employees’ assessments of their immediate supervisors are therefore particularly relevant for understanding how ethical leadership is experienced in organisational life (Kaluza et al., 2022). Employees who perceive their supervisors as ethical tend to view managerial decisions as fairer and more predictable, develop greater trust in their supervisors, and experience a stronger sense of relational security at work (Treviño et al., 2000; Palanski & Yammarino, 2009). Accordingly, ethical leadership has been associated with a range of favourable employee outcomes, including job satisfaction, work engagement, organisational commitment, and lower withdrawal tendencies (Lambert et al., 2024).

Nevertheless, the predominant emphasis on the favourable employee outcomes of ethical leadership may create the impression that this form of leadership is experienced uniformly across employees (Bartels & Wellman, 2023). Evaluating a supervisor as fair, principled, and ethical, however, does not necessarily imply that all aspects of an employee’s work experience are equally positive (De Hoogh & Den Hartog, 2008). Ethical leaders may establish clear behavioural standards, expect employees to take responsibility, and reinforce accountability. Although these characteristics can foster perceptions of trust and fairness, they do not necessarily eliminate job demands, time pressure, workload, or other sources of strain that employees may encounter (Lambert et al., 2024).

This distinction suggests that examining the relationship between ethical leadership and employee outcomes solely in terms of average effects may provide an incomplete picture. Employees reporting similarly favourable perceptions of ethical leadership may nevertheless differ substantially in their broader work experiences (McKenna & Jeske, 2021). For some employees, favourable leadership perceptions may coincide with low stress and low turnover intention, whereas for others, similarly favourable evaluations may coexist with substantial strain or strong intentions to leave (Lambert et al., 2024). The meaning of ethical leadership for employees may therefore be better understood not only through a linear logic in which “more ethical leadership leads to more favourable outcomes,” but also by considering how leadership perceptions combine with employees’ other organisational and psychological experiences (Wang & Xu, 2019). Such an approach does not challenge the established benefits of ethical leadership; rather, it raises the question of how uniformly these benefits are experienced across employees.

At the same time, employees’ work experiences extend beyond their evaluations of their immediate supervisors. While employees form perceptions of their supervisors’ ethical conduct, they also develop broader judgements about the extent to which the organisation values their contributions and supports their well-being (Kaluza et al., 2022). Perceptions of supervisory ethical leadership and organisational support therefore represent related but conceptually distinct aspects of the employee experience. Distinguishing between these two forms of evaluation is important for understanding when favourable leadership perceptions coincide with low strain and limited withdrawal tendencies, and when employees may experience stress or consider leaving despite evaluating their supervisors positively (Yue et al., 2022). The following section therefore considers perceived organisational support as a complementary element reflecting employees’ broader evaluations of their organisational environment.

### 2.2. Perceived Organisational Support in the Employee Experience

Perceived organisational support (POS) refers to employees’ general belief regarding the extent to which the organisation values their contributions and cares about their well-being (Kaluza et al., 2022). As an important indicator of the social exchange relationship between employees and their organisation, POS has been associated with favourable attitudes such as organisational commitment, job satisfaction, and work engagement, while generally showing negative associations with adverse employee outcomes such as job stress and turnover intention (Wang & Xu, 2019). POS therefore reflects not only employees’ relationships with a particular supervisor but, more broadly, their experience of being valued and supported by the organisation.

Although ethical leadership and POS are related, they represent distinct dimensions of the employee experience with different referents. Ethical leadership concerns employees’ evaluations of the behaviour of their immediate supervisor, whereas POS refers to the organisation itself. Nevertheless, because employees often view supervisors as representatives of the organisation, supervisors’ fair, trustworthy, and responsive behaviour may contribute to broader perceptions that the organisation values and supports its employees (Wang & Xu, 2019). Favourable perceptions of ethical leadership and high POS may therefore occur together. This conceptual proximity, however, does not imply that the two constructs are interchangeable or that they necessarily coincide with the same employee outcomes (Huang et al., 2021; Rodgers et al., 2024).

This distinction becomes particularly relevant when considering employee strain and withdrawal. High POS may provide an organisational resource that helps employees cope with work demands, while ethical leadership may constitute a more proximal social resource grounded in the quality of the supervisor–employee relationship (Rodgers et al., 2024; Shangguan et al., 2017). Yet perceiving both the supervisor as ethical and the organisation as supportive does not necessarily mean that pressures or stressors arising from other aspects of work are absent. Conditions such as workload, time pressure, shift work, and operational demands may remain part of employees’ work experiences despite favourable evaluations of both their supervisor and organisation (Eisenberger et al., 2020). Ethical leadership and POS may therefore represent important resources for employees without necessarily being accompanied by similarly low levels of stress or turnover intention across all employees.

Considering ethical leadership and POS jointly thus offers analytical value beyond examining their separate associations with employee outcomes (Huang et al., 2021). Bringing employees’ evaluations of their immediate supervisors and their broader organisational environment into the same framework makes it possible to examine how favourable social and organisational resources combine with experiences of strain and withdrawal. In particular, examining whether employees with similarly favourable perceptions of leadership and organisational support differ in their levels of stress and turnover intention moves beyond the assumption that employee experiences are homogeneous (Eisenberger et al., 2020). Rather than questioning the relevance of ethical leadership or organisational support, this perspective focuses on how these resources combine with other aspects of employees’ work experiences within distinct employee profiles.

### 2.3. Employee Job Stress and Turnover Intention

Job stress represents an important work experience that arises when employees perceive job demands as taxing or exceeding the resources available to them (Bartels & Wellman, 2023). Prolonged or high levels of job stress have been associated with a range of adverse outcomes, including burnout, lower job satisfaction, psychological strain, and withdrawal from the organisation (Liu et al., 2016). Among these outcomes, turnover intention is particularly important. Turnover intention refers to an employee’s conscious inclination to voluntarily leave the current organisation and is commonly regarded as one of the most proximal cognitive antecedents of actual turnover behaviour (Bartels & Wellman, 2023). Research suggests that as job stress increases, employees may perceive the costs of maintaining their current employment relationship as greater and become more inclined to consider alternative employment opportunities (Shangguan et al., 2017).

High job stress and turnover intention, however, do not necessarily arise from negative evaluations of the supervisor or the organisation. Employees’ work experiences are shaped simultaneously by multiple social, organisational, and job-specific conditions (Liu et al., 2016). An employee may perceive a supervisor as fair and ethical and believe that the organisation is supportive, while still experiencing substantial stress due to workload, time pressure, shift arrangements, or other operational demands. Similarly, considering leaving the organisation does not necessarily indicate low commitment or a negative evaluation of the supervisor (Griffeth et al., 2000). Turnover intention may emerge as a response to high strain, but it may also coexist with employees’ considerations regarding career development and alternative opportunities (Lyubykh et al., 2022). Treating stress and turnover intention simply as opposites of favourable perceptions of ethical leadership and organisational support may therefore provide an incomplete account of the complexity of employee experience (Mobley, 1977).

This distinction is particularly relevant to the literature that conceptualises ethical leadership and perceived organisational support as potentially protective resources for employees. Although both constructs have been associated with more favourable employee outcomes, these associations do not imply that employees experience similar organisational conditions in the same way (Huang et al., 2021). Some employees may combine favourable perceptions of their supervisor and organisation with low stress and low turnover intention, whereas others may experience substantial strain or consider leaving despite similarly favourable evaluations (Shangguan et al., 2017). The relevant question, therefore, extends beyond whether ethical leadership or organisational support is associated, on average, with lower stress and turnover intention; it also concerns the configurations in which these experiences coexist at the employee level.

Conceptualising job stress and turnover intention in this way requires viewing employee experience not simply as a set of independent variables but as a configuration of related experiences that may combine in different ways (Liu et al., 2016). Similar levels of favourable leadership and organisational support may coexist with different patterns of stress and withdrawal across employees (Griffeth et al., 2000). This possibility calls for moving beyond variable-centred approaches focused on average associations to examine whether distinct subgroups of employees are characterised by different combinations of ethical leadership, organisational support, job stress, and turnover intention. The following section therefore adopts a person-centred perspective to consider how these employee experiences may be configured jointly.

### 2.4. A Person-Centred Perspective on Employee Experiences

Research on ethical leadership, perceived organisational support, job stress, and turnover intention suggests that these constructs capture distinct aspects of employee experience and are meaningfully related to one another (Urbanaviciute & Lazauskaite-Zabielske, 2023). However, because these relationships have predominantly been examined through variable-centred approaches, less attention has been paid to the possibility that employees may experience them in different combinations. An approach focused on average associations may, for example, reveal a weak overall relationship between ethical leadership and job stress while obscuring the possibility that employees with similarly favourable perceptions of their supervisors differ substantially in their levels of stress (Bouckenooghe et al., 2019). Understanding heterogeneity in employee experiences therefore requires moving beyond the independent effects of individual variables to consider how these experiences combine within the same individual.

A person-centred perspective recognises that employees need not constitute a homogeneous group and that distinct subgroups may be characterised by different configurations of organisational and psychological experiences (Cortés-Denia et al., 2024; Urbanaviciute & Lazauskaite-Zabielske, 2023; Urick, 2016). From this perspective, ethical leadership and perceived organisational support represent two distinct evaluations of employees’ social and organisational environments, whereas job stress and turnover intention capture different aspects of strain and withdrawal. These experiences need not necessarily move in the same direction (Yue et al., 2022). For some employees, favourable leadership perceptions and high organisational support may coincide with low stress and low turnover intention, whereas for others, favourable contextual evaluations may coexist with substantial strain or intentions to leave. Identifying such patterns makes it possible to move beyond the assumption that favourable organisational resources are accompanied by the same experiential outcomes for all employees.

Career planning adds an exploratory dimension to this configuration by capturing employees’ future-oriented career orientation. Whereas turnover intention reflects an inclination to leave the current organisation, career planning reflects the extent to which employees actively engage with their own professional development and future careers. These tendencies are not necessarily opposite (Gillet et al., 2019). An employee may experience high levels of stress or consider leaving the current organisation while continuing to invest actively in career development. Considering career planning alongside the other indicators may therefore provide additional insight into whether withdrawal intentions coexist with continued career orientation rather than reflecting broader disengagement from one’s career (Bouckenooghe et al., 2019; Cortés-Denia et al., 2024). In the present study, however, career planning is treated not as a central component of the theoretical model but as an exploratory indicator that may contribute to the interpretation of employee profiles.

Accordingly, examining ethical leadership, perceived organisational support, job stress, turnover intention, and career planning jointly may provide a more differentiated understanding of how employee experiences are configured within an organisation. Rather than retesting whether ethical leadership or organisational support is beneficial on average, this approach focuses on the employee experiences with which these favourable evaluations coexist and on whether distinct employee profiles emerge under similarly favourable conditions (Griffeth et al., 2000). In doing so, a person-centred perspective makes it possible to examine whether favourable perceptions of leadership and organisational support necessarily coincide with low strain, thereby providing a more nuanced account of heterogeneity in employee experiences.

## 3. Methodology

### 3.1. Design and Setting

A cross-sectional survey design was used. Data were collected between April and June 2024 from employees of a single manufacturing organisation operating in Türkiye. All participants were shift workers drawn from four operational units: field production, production preparation, quality and maintenance.

A cross-sectional design is appropriate to the research questions, which concern the configuration of employee experiences at a point in time rather than change or causal sequence. Profile enumeration requires only that all indicators be measured contemporaneously. The design nonetheless places firm limits on interpretation: no temporal ordering among the variables can be established, and the associations reported below are not evidence of causal influence in either direction. This constraint is reflected in the language used throughout the results and discussion and is revisited in the limitations section.

### 3.2. Participants and Recruitment

The sampling frame comprised all shift employees of the participating organisation who had been in post for at least six months. Participation was voluntary and unpaid; employees were invited through internal channels and completed the questionnaire anonymously. Informed consent was obtained before the first item. Two hundred and forty-seven employees returned usable questionnaires with no missing responses on any scale item. Because all respondents selected the questionnaire’s 0–1-year tenure category and eligibility required at least six months of service, the available data place respondents between six and twelve months of organisational tenure; exact tenure in months was not recorded.

A survey response rate could not be calculated because the organisation did not record how many eligible employees received or accessed the invitation.

The sample is a single-organisation convenience sample, and this has three consequences that bear on interpretation. First, participation was self-selected, so employees with strong views about their supervisor or their workload may be over-represented relative to the indifferent. Second, all respondents are drawn from one employer, so organisational culture, management practice and sector conditions are constant and cannot be examined as sources of variation; conversely, differences between profiles cannot be attributed to differences in employer. Third, all participants recorded organisational tenure in the shortest available band, which reflects a period of substantial recruitment at the site. Given the six-month eligibility threshold, this band corresponds to six to twelve months of service, although precise tenure in months is unavailable. The sample is therefore a short-tenure, shift-based manufacturing workforce, and generalisation beyond comparable settings is not warranted.

Table 1 reports the demographic and occupational characteristics of the sample. These characteristics are presented in the Methods because they describe the sample rather than constituting a study finding.

### 3.3. Measures

All items were rated on a five-point Likert scale (1 = strongly disagree, 5 = strongly agree) and administered in Turkish. Scale scores are unweighted item means.

Ethical Leadership (EL). Ethical leadership was measured using a 4-item scale adapted from the Ethical Leadership Scale (Brown et al., 2005). Participants rated the conduct of their immediate supervisor. Internal consistency was high (α = 0.90); corrected item–total correlations ranged from 0.73 to 0.80.

Job Stress (ST). Job stress was assessed using a 9-item scale based on the Job Stress Scale (Parker & DeCotiis, 1983), covering workload, time pressure and job uncertainty. Internal consistency was high (α = 0.89); corrected item–total correlations ranged from 0.56 to 0.74.

Perceived Organisational Support (POS). POS was measured using a 5-item scale derived from the Survey of Perceived Organizational Support (Eisenberger et al., 1986). Internal consistency was high (α = 0.87); corrected item–total correlations ranged from 0.61 to 0.77.

Turnover Intention (JT). Turnover intention was assessed using a 5-item scale adapted from the Turnover Intention Scale (Mobley, 1977). Internal consistency was acceptable (α = 0.80); corrected item–total correlations ranged from 0.42 to 0.75.

Career Planning (CP). Career planning was assessed using a 14-item study-specific measure covering perceived developmental opportunities and career structure within the organisation (Zhang et al., 2023). One item was negatively worded and reverse-scored before analysis. Internal consistency was high (α = 0.86). Corrected item–total correlations ranged from 0.26 to 0.71; the reverse-scored item was the weakest performer. Because this measure does not derive from an established published scale, its contribution to the profile solution is reported separately and interpreted with corresponding caution.

The administered questionnaire contained only the five measures described above (37 items in total). No subjective value-congruence instrument was administered, and no value-congruence variable is included in any analysis reported in this study.

### 3.4. Analytic Strategy

Because all five indicators are continuous scale scores, the mixture model estimated here is a latent profile analysis, the continuous-indicator form of latent class analysis (Bauer, 2022). Models with one to six profiles were estimated on standardised indicators using Gaussian mixtures with diagonal covariance structure and 30 random starts per solution to reduce the risk of local maxima.

Model selection followed established practice (Killian et al., 2019) and combined four criteria: information criteria (AIC, BIC, sample-size-adjusted BIC), a bootstrap likelihood-ratio test comparing k − 1 with k profiles, classification quality (entropy and average posterior probabilities), and substantive interpretability together with the requirement that no profile fall below 5% of the sample (Urbanaviciute & Lazauskaite-Zabielske, 2023). No single index was treated as decisive.

Following profile enumeration, differences between profiles on each indicator were tested with one-way analysis of variance and eta-squared effect sizes, and associations between profile membership and categorical demographic variables were tested with chi-square and Cramér’s V (Bauer, 2022; Lyubykh et al., 2022).

### 3.5. Common Method Variance

All measures were self-reported by the same respondents at a single time point, so common method variance is a genuine concern. Harman’s single-factor test was conducted by submitting all 37 items to an unrotated principal component analysis. Eight components had eigenvalues above 1.0, and the first accounted for 18.5% of the total variance, well below the 50% threshold conventionally taken to indicate a problem (Bouckenooghe et al., 2019; Cortés-Denia et al., 2024). This test is a weak diagnostic and its result should not be read as demonstrating the absence of method bias, only as failing to detect a dominant general factor. The pattern of correlations reported below provides indirect additional evidence: job stress is essentially uncorrelated with ethical leadership and perceived organisational support, which would be difficult to reconcile with a strong general response tendency inflating all associations.

## 4. Results

### 4.1. Descriptive Statistics and Correlations

Table 2 reports means, standard deviations, reliabilities and intercorrelations. Ethical leadership was moderately positive (M = 3.47, SD = 0.99), as were perceived organisational support (M = 3.37, SD = 0.88) and career planning (M = 3.67, SD = 0.65). Job stress (M = 3.08, SD = 0.91) and turnover intention (M = 3.31, SD = 0.89) were moderate. All distributions were approximately symmetric (|skew| ≤ 0.46, |kurtosis| ≤ 0.59).

Two features of the correlation matrix are central to what follows. First, job stress is essentially unrelated to ethical leadership (r = 0.02, *p* = 0.820) and to perceived organisational support (r = 0.04, *p* = 0.492). Second, job stress is substantially related to turnover intention (r = 0.43, *p* < 0.001), while ethical leadership is not (r = −0.08, *p* = 0.207) and perceived organisational support only weakly so (r = −0.13, *p* = 0.040). At the variable-centred level, therefore, job stress shows a substantially stronger association with turnover intention than either ethical leadership or perceived organisational support.

### 4.2. Profile Enumeration

Table 3 reports fit statistics for one- to six-profile solutions. AIC declined monotonically and reached its lowest value for the six-profile solution (3279.48). Sample-size-adjusted BIC also reached its lowest value for six profiles (3301.54), whereas BIC reached its minimum at four profiles (3450.20). The bootstrap likelihood-ratio test was significant for each comparison up to four profiles (3 vs. 4: LRT = 63.16, *p* = 0.010) but was non-significant for the five-profile solution (4 vs. 5: LRT = 25.84, *p* = 0.079). Thus, the information criteria provided mixed evidence: AIC and adjusted BIC favoured a more complex solution, while BIC and the bootstrap test supported retaining four profiles.

The four-profile solution was retained based on the convergence of BIC, the bootstrap likelihood-ratio test, classification quality, profile size and substantive interpretability rather than on the minimum of every information criterion. Its entropy (0.845) exceeded the 0.80 benchmark for acceptable classification, average posterior probabilities ranged from 0.877 to 0.928, and the smallest profile comprised 9.7% of the sample. Although the three-profile solution had marginally higher entropy (0.897), the bootstrap test supported the improvement from three to four profiles, and the three-profile solution merged groups that differed markedly in perceived organisational support (1.86 vs. 3.32). Conversely, adding a fifth profile reduced entropy to 0.761, increased BIC, and was not supported by the bootstrap test. The four-profile solution was therefore judged to provide the most defensible balance between fit, parsimony, classification quality and substantive distinctiveness.

### 4.3. Profile Description

Table 4 reports profile means and within-profile standard deviations. Profiles are ordered by level of ethical leadership.

Profile 1—Unsupported (13.4%). This group is defined primarily by very low perceived organisational support (1.86), the lowest value on any indicator in any profile. Ethical leadership is also low (2.65) and highly variable (SD = 1.36), the largest within-profile dispersion observed. Job stress is close to the sample mean (3.21) and turnover intention is elevated (3.62). These employees report moderate job stress but describe an organisation they do not experience as supportive.

Profile 2—Moderate (60.7%). The numerically dominant group reports mid-range values across all five indicators, with the lowest job stress in the sample after Profile 4 (2.80). This profile represents the modal experience in this workforce and provides the reference against which the smaller profiles are interpreted.

Profile 3—Strained (16.2%). This group reports high ethical leadership (4.11) and high perceived organisational support (4.09), together with the highest job stress in the sample (4.37) and the highest turnover intention (4.10). Within-profile dispersion on job stress is very low (SD = 0.29), indicating that this is a consistent rather than an averaged pattern. Career planning is also high (3.98). These employees evaluate both supervisor and organisation favourably while reporting substantial strain and a strong intention to leave.

Profile 4—Thriving (9.7%). This group reports the highest ethical leadership (4.65) and the highest perceived organisational support (4.57), with low job stress (2.48) and the lowest turnover intention in the sample (2.18). Career planning is high (3.99).

The comparison between Profiles 3 and 4 is the central result. The two groups differ by 0.54 on ethical leadership and 0.48 on perceived organisational support, modest gaps at the upper end of both scales, yet by 1.89 on job stress and 1.92 on turnover intention. Both groups evaluate their supervisor and their organisation favourably; one is under considerable strain and intends to leave, the other is not. Favourable evaluations of leadership and organisational support therefore do not consistently correspond to low strain and withdrawal across profiles in this sample.

This pattern provides additional context for interpreting the near-zero bivariate correlation reported in Table 2. Profiles 3 and 4 both occupy the upper range of ethical leadership while differing markedly in job stress. The person-centred analysis therefore reveals heterogeneity that is not apparent from the bivariate association alone. Rather than demonstrating that the near-zero correlation is an artefact of aggregation, these findings indicate that similar levels of favourable leadership evaluation may coexist with substantially different experiences of strain.

### 4.4. Profiles and Demographic Characteristics

Profile membership was associated with organisational unit (χ^2^ = 26.32, df = 9, *p* = 0.002, V = 0.19), age (χ^2^ = 18.76, df = 9, *p* = 0.027, V = 0.16), position (χ^2^ = 14.65, df = 6, *p* = 0.023, V = 0.17) and gender (χ^2^ = 9.53, df = 3, *p* = 0.023, V = 0.20). Effect sizes are small in every case. No association was found with education (*p* = 0.431), marital status (*p* = 0.288) or self-reported general motivation (*p* = 0.411). Given the number of tests conducted and the modest effect sizes, these associations should be treated as descriptive and require replication before any weight is placed on them.

## 5. Discussion

This study asked whether favourable evaluations of supervisory ethical leadership are consistently accompanied by lower strain and weaker turnover intentions. In this workforce they are not. Four profiles were identified, two of which report high ethical leadership and high perceived organisational support while differing sharply in job stress and turnover intention.

### 5.1. Heterogeneity Rather than Absence of Effect

The near-zero correlation between ethical leadership and job stress (r = 0.02) indicates little aggregate association between the two variables in this sample. The profile analysis, however, provides additional information about heterogeneity in employee experiences. Employees who rate their supervisors favourably are represented in profiles characterised by markedly different levels of strain. Rather than supporting a uniform relationship between ethical leadership and job stress, the findings suggest that favourable leadership evaluations may coexist with different levels of strain across employees, potentially reflecting contextual or individual factors not captured in the present design. This has a methodological implication that extends beyond the present case (Killian et al., 2019). Null findings in leadership research are ordinarily interpreted as evidence of no relationship. Where a sample contains subgroups with opposing configurations, a null correlation is instead a signal that the sample is not homogeneous with respect to the relationship being estimated. Person-centred analysis provides a means of distinguishing these two situations, and the distinction cannot be drawn from the correlation alone (Hølge-Hazelton et al., 2021).

### 5.2. Interpreting the Strained Profile

The Strained profile is the most theoretically interesting group and also the one most open to competing explanations. Three interpretations are consistent with the data, and the present design cannot adjudicate between them.

One possible explanation is that employees in the Strained profile are exposed to job demands that are not captured by the leadership and support measures included in this study. Profile membership differed across organisational units, suggesting that work context may contribute to the observed configuration. In a shift-based manufacturing setting, factors such as workload, production pressure, scheduling demands, or physical demands may generate strain even when employees evaluate their supervisors and the organisation favourably (Brewer et al., 2024). However, because these demands were not directly measured, this interpretation remains provisional. The low within-profile variance in job stress (SD = 0.29) indicates that high strain is a relatively consistent characteristic of this group, but it does not establish its source.

A second possibility concerns career orientation. The relatively high career-planning scores observed in the Strained profile may indicate that some of these employees remain actively oriented toward their future development despite experiencing substantial strain. Under this interpretation, turnover intention need not reflect a negative evaluation of the supervisor or organisation; it may coexist with an active search for career development or alternative opportunities (Zhang et al., 2023). Given the exploratory nature of the career-planning measure, however, this interpretation should remain tentative.

A third possibility is that individual differences in affective disposition contribute to the profile. Employees higher in negative affectivity may report greater strain across work contexts. Yet this explanation alone appears insufficient, because the same employees also report favourable ethical leadership and organisational support. Future studies incorporating dispositional measures would be required to evaluate this possibility directly.

We note explicitly that we cannot distinguish between these accounts. Doing so would require measurement of objective job demands, longitudinal data, or both. What the data establish is that high evaluations of leadership and support coexist with high strain in a substantial and internally consistent subgroup. This indicates that favourable leadership evaluations alone are insufficient for identifying employees who report high strain; it does not establish that leadership improvement would fail to reduce strain (Hølge-Hazelton et al., 2021).

### 5.3. The Unsupported Profile

Profile 1 is defined by very low perceived organisational support alongside moderate job stress. Its most distinctive measured characteristic is not elevated job stress but very low perceived organisational support. Turnover intention is elevated (3.62), but less so than in the Strained profile, suggesting that elevated turnover intention may occur in qualitatively different experiential configurations (Mobley, 1977). The wide dispersion in ethical leadership within this profile (SD = 1.36) indicates that low organisational support is reported by employees with quite different views of their immediate supervisor, which supports the conceptual separation of the two constructs set out in Section 2.2. Supervisory conduct and organisational support are evaluated independently by these employees.

### 5.4. Theoretical Implications

The findings contribute to ethical leadership research by challenging the assumption that favourable perceptions of ethical leadership are accompanied by uniformly favourable employee experiences. The results do not suggest that ethical leadership is ineffective or detrimental. Rather, they indicate that similarly positive evaluations of supervisory ethical leadership can coexist with markedly different levels of employee strain and withdrawal (Wang & Xu, 2019). The contrast between the Strained and Thriving profiles is particularly informative in this regard. Both groups evaluated their supervisors and the organisation favourably, yet they differed substantially in job stress and turnover intention. This pattern suggests that the consequences associated with ethical leadership are unlikely to be uniform across employees and that favourable leadership perceptions should not be treated as sufficient indicators of a broadly positive employee experience (Griffeth et al., 2000).

A second contribution concerns the distinction between supervisory and organisational evaluations. Ethical leadership and perceived organisational support are often positively associated, but they refer to different targets within the employee’s work environment (Brown & Treviño, 2006). The profile structure observed here reinforces this distinction. In the Unsupported profile, perceived organisational support was consistently low, whereas evaluations of ethical leadership displayed considerable within-profile variation. This pattern indicates that employees may evaluate their immediate supervisor and the broader organisation differently, even within the same work context. Conceptually, this supports treating ethical leadership and perceived organisational support as related but non-interchangeable aspects of the employee experience rather than as manifestations of a single general positive evaluation.

Third, the study demonstrates the theoretical value of examining heterogeneity in leadership research through a person-centred lens. At the aggregate level, ethical leadership was essentially unrelated to job stress. A variable-centred interpretation might therefore conclude that ethical leadership has little relevance for employee strain. The profile analysis reveals a more complex structure: employees with similarly high evaluations of ethical leadership were distributed across subgroups with sharply different levels of stress and turnover intention (Lambert et al., 2024). Aggregate associations may therefore conceal qualitatively different configurations of employee experience when opposing patterns coexist within the same sample. This finding suggests that weak or null relationships in leadership research should not automatically be interpreted as evidence of homogeneity or absence of meaningful structure.

Taken together, these findings shift attention from whether ethical leadership or organisational support is beneficial on average to how favourable supervisory and organisational evaluations are configured with strain and withdrawal at the employee level. The theoretical implication is not that positive leadership and organisational support cease to matter, but that their meaning depends on the broader configuration in which they are experienced (Lambert et al., 2024). A configurational perspective therefore offers a more differentiated account of employee responses by recognising that similar positive contextual evaluations may accompany substantially different psychological and behavioural orientations.

### 5.5. Practical Implications

The following suggestions follow from the profile structure, but the design is cross-sectional and single-organisation, and none has been tested as an intervention. They should be read as hypotheses for practice rather than as evidence-based recommendations.

The principal implication is that leadership development and demand management address different problems. In this workforce, the group with the highest reported turnover intention also expressed a highly favourable view of its supervisor. Further investment in supervisory training alone may therefore be insufficient for this group, and organisations may need to examine other aspects of work design, including workload, scheduling, and staffing (Kim & Beehr, 2018). Organisations that monitor leadership quality as a proxy for retention risk may therefore overlook employees who report elevated intentions to leave.

A second implication concerns diagnosis. Because the Strained and Thriving profiles both report high ethical leadership and perceived organisational support, aggregate reporting of those measures obscures their pronounced difference in strain and turnover intention. Separate reporting of strain indicators, and attention to their distribution rather than only their mean, would surface this distinction. The finding that profile membership varies by organisational unit suggests that unit-level rather than organisation-level reporting may be more informative, although this proposition requires prospective evaluation.

A third implication concerns the Unsupported profile, whose defining feature is low perceived organisational support rather than high strain. Interventions directed at workload would not address this group; recognition, communication and evidence of organisational investment in the individual would be more closely matched to what these employees report.

### 5.6. Limitations

Several limitations constrain the conclusions. The study is cross-sectional. No temporal ordering can be established among the variables, and no causal claim is made or supported. The profiles describe co-occurring patterns at a single point in time and may not be stable. All variables were reported by the same individuals in one questionnaire. Harman’s test did not indicate a dominant general factor, but this is a weak diagnostic. Future work should separate sources or introduce temporal separation between measurements. The sample comprises 247 shift employees of one manufacturing organisation in Türkiye, all within the questionnaire’s 0–1-year tenure category. Because eligibility required at least six months of service, the available range was six to twelve months, but exact tenure in months was not collected. Sector, organisational size, ownership, national culture and tenure range are therefore constrained, and the findings should not be extended to other sectors, to salaried or non-shift work, or to longer-tenured employees without replication. The uniform short-tenure range is a particular constraint: profile membership may reflect a stage of organisational entry rather than a stable configuration. Participation was voluntary within a single organisation, so self-selection cannot be excluded. Employees with strong views may be over-represented, which could inflate the size of the extreme profiles relative to the workforce as a whole. Ethical leadership was assessed with a four-item scale, which limits coverage of the construct’s dimensionality. The career planning scale is the least established of the five instruments used; its weakest item was the single reverse-worded item, and its contribution to the profile solution was the smallest of any indicator (η^2^ = 0.10). Findings involving career planning should be regarded as provisional. Objective job demands, staffing levels, shift patterns and labour market conditions were not measured. These are plausible explanations for the Strained profile and their absence is the principal limitation on the interpretation offered in Section 5.2.

### 5.7. Future Research

Four directions follow directly. First, longitudinal designs would establish whether profile membership is stable and whether transitions between profiles predict actual turnover as distinct from intention; latent transition analysis is the natural extension. Second, future studies should measure competing explanations for the Strained profile in the same design: objective and perceived job demands (including workload, staffing, scheduling and physical demands), external career opportunities and career orientation, negative affectivity, and unit- or shift-level conditions. Including these variables would permit direct comparison of demand-based, career-based, dispositional and contextual accounts rather than relying on post hoc interpretation. Third, replication in other sectors and across precisely measured tenure bands would establish whether the four-profile structure is specific to short-tenure shift manufacturing. Fourth, multi-source designs separating the rater of leadership from the reporter of strain would address the common method concern directly.

## 6. Conclusions

This study used latent profile analysis to examine how employee-rated ethical leadership, job stress, perceived organisational support, turnover intention and career planning co-occurred among 247 shift-working employees in a Turkish manufacturing organisation. Four profiles were identified: Unsupported, Moderate, Strained and Thriving. These profiles demonstrate that favourable assessments of a supervisor and the organisation do not necessarily coincide with low strain or weak intentions to leave.

The central finding was the contrast between the Strained and Thriving profiles. Both occupied the upper ranges of ethical leadership and perceived organisational support, yet they differed sharply in job stress and turnover intention. This configurational difference complements the near-zero full-sample correlations between job stress and both ethical leadership and organisational support, showing that aggregate associations can conceal substantively different employee experiences. The results also indicate that strain-related withdrawal may reflect job demands that are not captured by leadership or support evaluations.

The specific contribution of the person-centred approach is therefore configurational rather than merely descriptive. A traditional variable-centred analysis establishes that ethical leadership has almost no average association with job stress in this sample; it does not reveal that employees occupying the favourable end of the leadership distribution separate into a high-strain/high-turnover-intention profile and a low-strain/low-turnover-intention profile. Identifying these coexisting configurations shows which employee experiences are combined within subgroups and prevents a near-zero average association from being interpreted as evidence of a uniform workforce.

The findings suggest that leadership development, organisational support and job-demand management should be treated as related but distinct areas of organisational practice. However, the cross-sectional, single-source and single-organisation design does not permit causal inference or broad generalisation. Replication across sectors, longitudinal assessment of profile stability and actual turnover, and the inclusion of objective work-demand indicators are required before the four-profile structure can be considered generalisable.

## Figures and Tables

**Table 1:** Demographic and occupational characteristics of participants (*N* = 247).

Characteristic	Category	*n*	%
Age	18–25	39	15.8
	26–34	72	29.1
	35–49	103	41.7
	50–64	33	13.4
Gender	Male	172	69.6
	Female	75	30.4
Education	High school	153	61.9
	Associate degree	60	24.3
	Bachelor’s degree	20	8.1
	Master’s degree	14	5.7
Marital status	Married	154	62.3
	Single	93	37.7
Organisational tenure	0–1 year (eligible ≥ 6 months; exact months not recorded)	247	100.0
Unit	Field production	93	37.7
	Production preparation	77	31.2
	Quality	43	17.4
	Maintenance	34	13.8
Position	Operative	135	54.7
	Foreman	66	26.7
	Machine operator	46	18.6
Work arrangement	Shift work	247	100.0

**Table 2:** Descriptive statistics, reliabilities and correlations (*N* = 247).

Variable	M	SD	α	1	2	3	4	5
1. Ethical leadership	3.47	0.99	0.90	—				
2. Job stress	3.08	0.91	0.89	0.02	—			
3. Perceived org. support	3.37	0.88	0.87	0.46 **	0.04	—		
4. Turnover intention	3.31	0.89	0.80	−0.08	0.43 **	−0.13 *	—	
5. Career planning	3.67	0.65	0.86	0.27 **	0.08	0.32 **	0.09	—

Note. * *p* < 0.05, ** *p* < 0.001. Job stress is not significantly correlated with ethical leadership (*p* = 0.820) or perceived organisational support (*p* = 0.492).

**Table 3:** Fit statistics for one- to six-profile solutions.

Profiles	LL	Par.	AIC	BIC	aBIC	Entropy	BLRT *p*	Smallest %
1	−1749.88	10	3519.77	3554.86	3523.16	—	—	100.0
2	−1683.34	21	3408.68	3482.37	3415.81	0.918	0.010	14.6
3	−1638.76	32	3341.53	3453.83	3352.39	0.897	0.010	9.3
4	−1606.65	43	3299.29	3450.20	3313.89	0.845	0.010	9.7
5	−1594.29	54	3296.58	3486.09	3314.91	0.761	0.079	6.1
6	−1574.74	65	3279.48	3507.59	3301.54	0.780	—	6.1

Note. LL = log-likelihood; Par. = number of free parameters; aBIC = sample-size-adjusted BIC; BLRT = bootstrap likelihood-ratio test comparing k − 1 with k profiles (100 replications). The four-profile solution was retained.

**Table 4:** Profile means and within-profile standard deviations.

Variable	P1 Unsupported (*n* = 33, 13.4%)	P2 Moderate (*n* = 150, 60.7%)	P3 Strained (*n* = 40, 16.2%)	P4 Thriving (*n* = 24, 9.7%)	F	η^2^
Ethical leadership	2.65 (1.36)	3.28 (0.80)	4.11 (0.48)	4.65 (0.37)	37.25 **	0.32
Job stress	3.21 (1.07)	2.80 (0.60)	4.37 (0.29)	2.48 (0.96)	61.89 **	0.43
Perceived org. support	1.86 (0.51)	3.32 (0.53)	4.09 (0.47)	4.57 (0.35)	171.78 **	0.68
Turnover intention	3.62 (0.76)	3.21 (0.74)	4.10 (0.58)	2.18 (1.01)	35.67 **	0.31
Career planning	3.38 (0.83)	3.60 (0.59)	3.98 (0.45)	3.99 (0.74)	8.50 **	0.10
Avg. posterior prob.	0.877	0.921	0.928	0.918	—	—

Note. ** *p* < 0.001. Standard deviations in parentheses. Entropy = 0.845.

## Data Availability

The data presented in this study are available from the corresponding author upon reasonable request. The data are not publicly available due to ethical and privacy considerations.
